# Transcatheter Mitral Valve Repair With MitraClip® in a Heart Transplant Recipient: Pushing the Limits of Percutaneous Valve Therapy

**DOI:** 10.7759/cureus.98795

**Published:** 2025-12-09

**Authors:** Pasquale Gagliardi, Marco Ferlini, Romina Frassica, Mauro Acquaro, Leonardo De Luca

**Affiliations:** 1 Division of Cardiology, Fondazione IRCCS Policlinico San Matteo, Pavia, ITA; 2 Department of Molecular Medicine, University of Pavia, Pavia, ITA

**Keywords:** case report, heart transplantation, left atrial thrombus, mitraclip, mitral regurgitation, percutaneous edge to edge repair

## Abstract

We report the case of a 62-year-old female who developed severe symptomatic functional mitral regurgitation (MR) 18 years after heart transplantation. Given her extremely high surgical risk, transcatheter edge-to-edge mitral valve repair with the MitraClip® device was selected as the most appropriate therapeutic option. The procedure was technically challenging due to altered post-transplant anatomy, but it was completed successfully without immediate complications and resulted in a substantial reduction in regurgitation. During early follow-up, a small left atrial thrombus was identified and subsequently resolved after a six-week course of oral anticoagulation. This report illustrates the feasibility and safety of transcatheter mitral repair in carefully selected heart transplant recipients and underscores the importance of tailored post-procedural surveillance in this complex population.

## Introduction

Mitral regurgitation (MR) in heart transplant recipients may result from annular dilation, graft dysfunction, or leaflet tethering [1]. Surgical correction is associated with substantial perioperative risk due to prior sternotomies, adhesions, and comorbidities related to chronic immunosuppression [2]. Transcatheter mitral valve repair with the MitraClip® system has emerged as a valuable alternative for patients with symptomatic severe MR who are considered prohibitive surgical candidates; however, published experience in the transplant population remains limited [3,4]. We describe a technically demanding but successful MitraClip® procedure in a long-term heart transplant recipient and discuss thrombotic surveillance and management considerations in this unique clinical setting.

## Case presentation

A 62-year-old female with a history of orthotopic heart transplantation in 2006 for chemotherapy-induced cardiomyopathy and maintained on routine post-transplant follow-up presented with progressive exertional dyspnea (New York Heart Association class III) and worsening MR on serial transthoracic echocardiograms. Her medical history included stage IV chronic kidney disease with a stable estimated glomerular filtration rate of 35 ml/min/1.73 m², prior endovascular aortic repair, and coronary stenting. Maintenance immunosuppression consisted of mycophenolate mofetil and everolimus. On March 15, 2024, transesophageal echocardiography (TEE) demonstrated preserved left ventricular ejection fraction (65%) and severe MR secondary to fibro-degenerative valve leaflet alterations, including posterior leaflet hypomobility and marked left atrial enlargement. Doppler indices suggested elevated left ventricular filling pressures without evidence of pulmonary hypertension.

**Figure 1:**
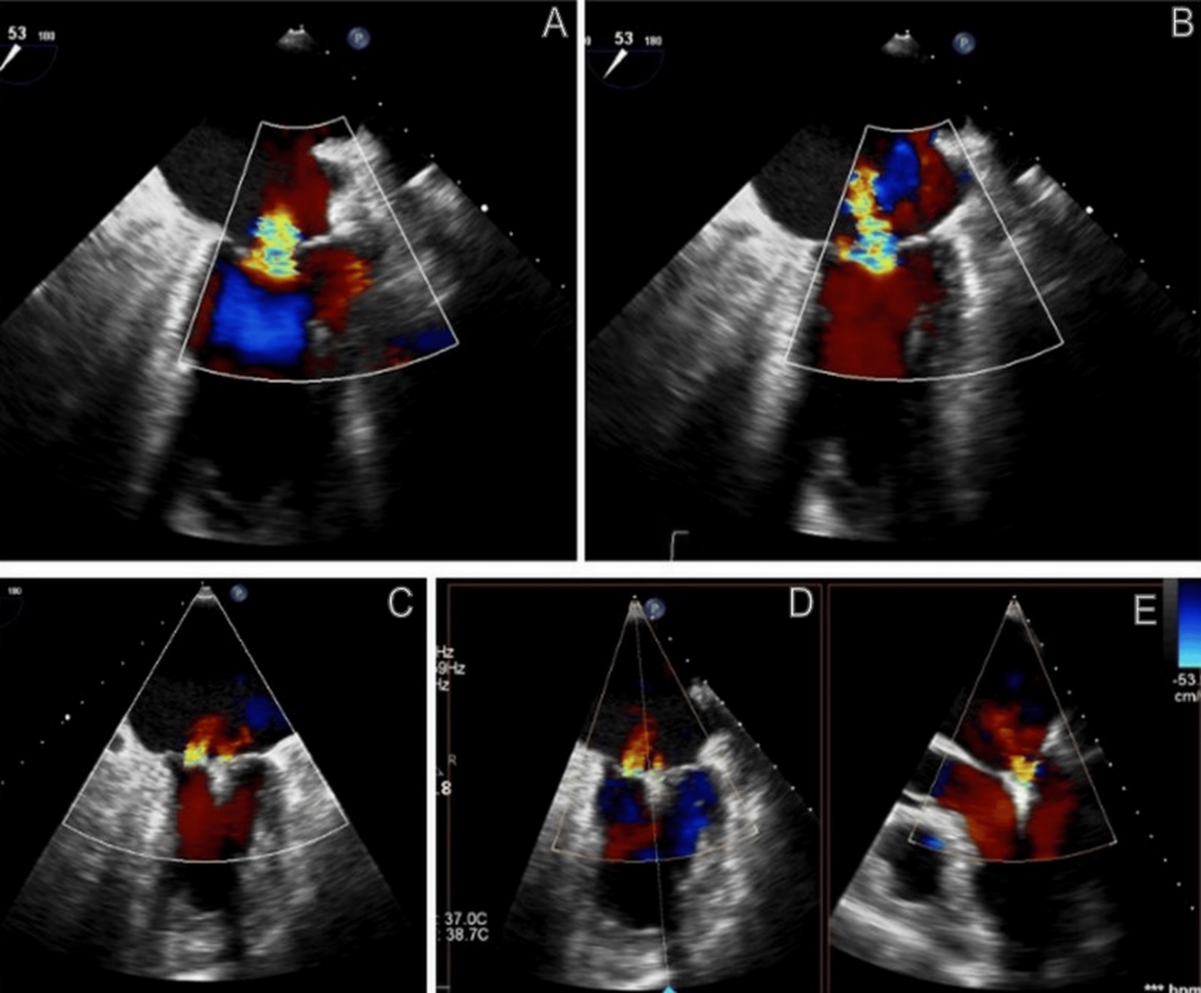
Color Doppler TEE (A, B) Color Doppler TEE showing severe mitral regurgitation before clip deployment. (C, D, E) Color Doppler TEE images demonstrating the reduction of mitral regurgitation after clip placement TEE: transesophageal echocardiography

**Figure 2:**
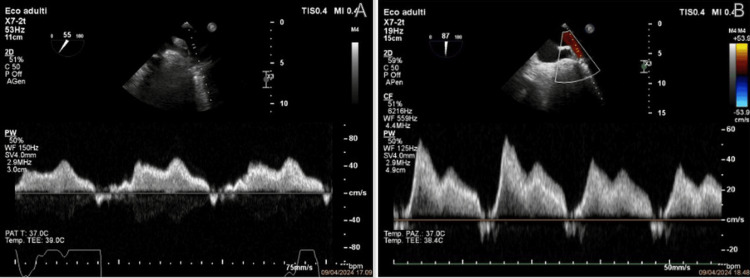
Pulmonary vein Doppler images (A) Pulmonary vein Doppler before MitraClip® showing systolic flow reversal. (B) Pulmonary vein Doppler after clip deployment demonstrating restoration of systolic-dominant flow

Following Heart Team evaluation, the patient was deemed high surgical risk, and transcatheter edge-to-edge repair (TEER) with the MitraClip system was planned. She was admitted on April 8, 2024, for periprocedural optimization, and slow intravenous hydration was initiated to minimize the risk of contrast-induced kidney injury. On April 9, after endotracheal intubation and general anesthesia, coronary angiography confirmed the absence of significant epicardial coronary artery disease. Under TEE guidance, transseptal access via the right femoral vein was attempted. Anatomical distortion from prior surgery made septal crossing and leaflet grasping challenging, requiring multiple attempts before successful advancement of the guide system into the left atrium. A single MitraClip XTW was deployed in a central-medial position (A2-P2), reducing MR to mild severity with a residual mean transmitral gradient of 3 mmHg and normalization of pulmonary venous systolic flow. (Figures 1, 2).

**Figure 3:**
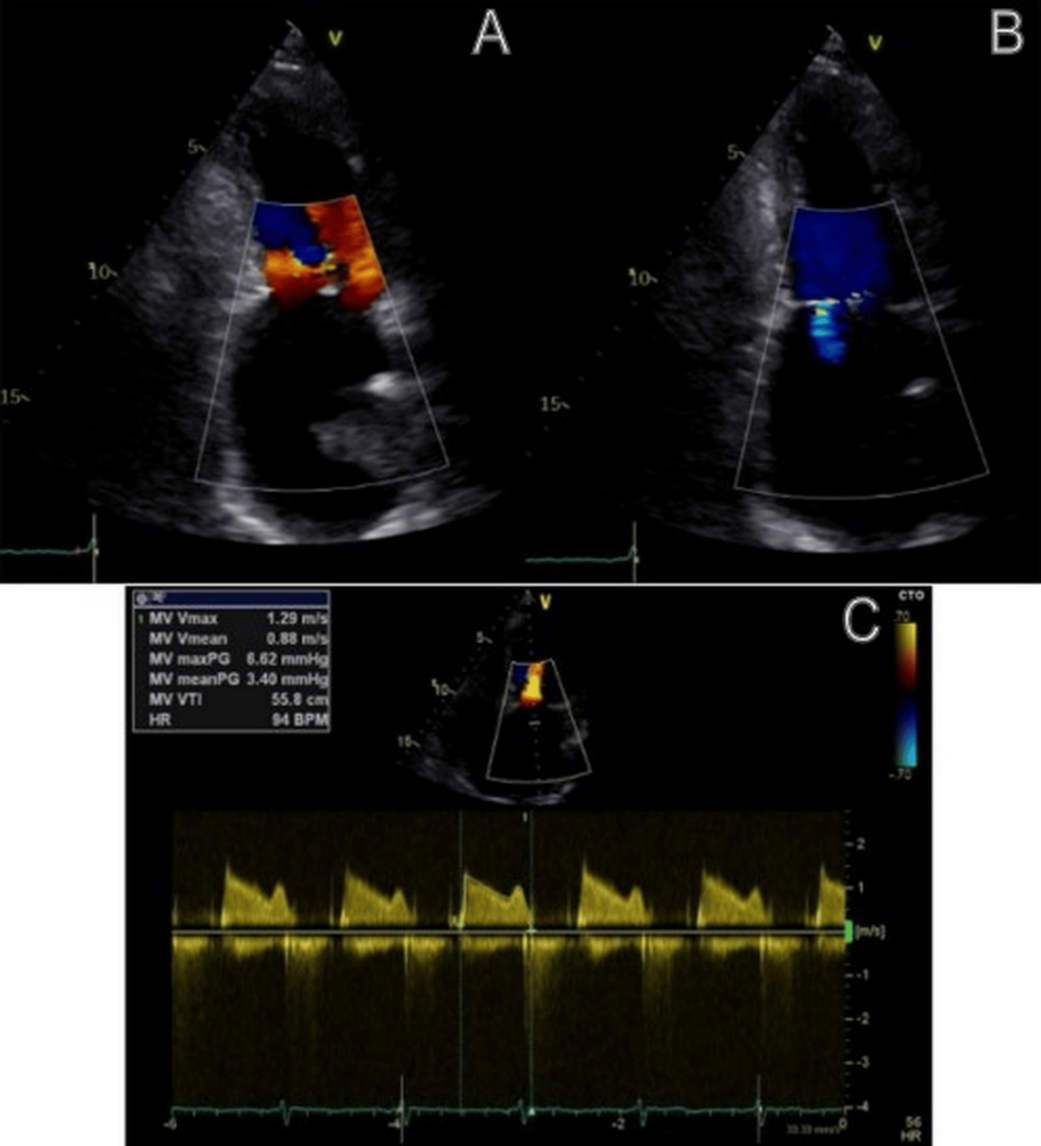
Follow-up imaging findings (A, B) Transthoracic echocardiogram at 1-year follow-up showing trivial mitral regurgitation. (C) Continuous-wave Doppler showing a mean transmitral gradient of 3.4 mmHg

The patient was extubated in the catheterization laboratory and transferred to the intensive cardiac care unit. During her stay, arterial blood gas analysis revealed metabolic acidosis due to bicarbonate depletion, which resolved promptly after intravenous administration of 70 mEq sodium bicarbonate. She was discharged on April 12 (postoperative day three). At the three-month follow-up, TEE identified a small mural left atrial thrombus adjacent to the clip. Warfarin was initiated with a target international normalized ratio of 2-3, resulting in complete thrombus resolution after six weeks. At nine months, transthoracic echocardiography demonstrated stable mild MR (mean gradient: 3.4 mmHg), preserved left ventricular ejection fraction, and no thromboembolic events. The patient reported excellent functional status (New York Heart Association class I) (Figure 3).

## Discussion

MR following heart transplantation is a recognized but challenging entity, often arising from a combination of annular dilatation, graft remodeling, leaflet tethering, and atrial dysfunction [1]. Its management can be particularly complex because surgical intervention carries substantial perioperative risk due to prior sternotomy, dense mediastinal adhesions, and comorbidities related to chronic immunosuppression [2]. Hence, TEER has progressively emerged as an appealing alternative for carefully selected high-risk patients, experience in the transplant population is still limited to small series and case reports [3,4]. 

As our case confirms, TEER in transplanted hearts may present several technical challenges. Altered atrial geometry, variable septal thickness and fibrosis, and changes in leaflet mobility can complicate both transseptal access and leaflet grasping. These anatomical aspects underline the importance of comprehensive preprocedural imaging and experienced operators. Despite these challenges, TEER has shown encouraging procedural success and symptomatic benefit in the few reports available, supporting its feasibility in this unique population. Another important aspect highlighted by our case is the predisposition of transplant recipients to left atrial thrombosis. Multiple factors - including atrial mechanical impairment, endothelial dysfunction, and immunosuppressive therapy - may contribute to a prothrombotic milieu [5].

The detection of a small mural thrombus during follow-up underscores the need for vigilant surveillance after TEER. While warfarin remains the standard treatment for device-related thrombi, the role of direct oral anticoagulants in this setting warrants further investigation. Overall, this report reinforces the concept that TEER may represent a valuable therapeutic option for heart-transplant recipients with severe MR who are not optimal candidates for surgical intervention. However, the limited body of literature highlights the need for further dedicated studies to better define patient selection, procedural strategies, and optimal antithrombotic regimens in this complex clinical environment.

## Conclusions

TEER with the MitraClip® system may represent a viable and effective option for selected heart-transplant recipients with severe functional MR who are not optimal candidates for surgical intervention. This report illustrates that, despite the anatomical challenges associated with the transplanted heart, TEER can achieve meaningful clinical improvement and provide a durable reduction in regurgitation. A multidisciplinary Heart Team approach, meticulous procedural planning, and vigilant post-procedural surveillance, particularly for thrombotic complications, are essential to optimize outcomes in this complex patient population.
